# A Novel Intelligent Approach to Lane-Change Behavior Prediction for Intelligent and Connected Vehicles

**DOI:** 10.1155/2022/9516218

**Published:** 2022-01-17

**Authors:** Luyao Du, Wei Chen, Jing Ji, Zhonghui Pei, Bingming Tong, Hongjiang Zheng

**Affiliations:** ^1^School of Automation, Wuhan University of Technology, Wuhan 430070, China; ^2^School of Information Engineering, Wuhan University of Technology, Wuhan 430070, China; ^3^Shanghai Engineering Technology Research Center for Intelligent and Connected Vehicle Terminals, Shanghai 200030, China; ^4^Shanghai PATEO Electronic Equipment Manufacturing Co., Ltd., Shanghai 200030, China

## Abstract

The prediction of lane-change behavior is a challenging issue in intelligent and connected vehicles (ICVs), which can help vehicles predict in advance and change lanes safely. In this paper, a novel intelligent approach, which considering both the driving style-based lane-change environment and the driving trajectory-related parameters of the ICV and surrounding vehicles, is proposed to predict the lane-change behaviors for ICVs. By analyzing the characteristics of the lane-change behavior of the vehicle, a modified dataset for the prediction of lane-change behavior was established based on the Next-Generation Simulation (NGSIM) dataset, which is made up of real vehicle trajectories collected by camera. In the proposed approach, the hidden Markov model (HMM)-based model is designed to judge whether the current environment is suitable for lane change according to the driving environment parameters around the vehicle; then according to the driving state of the vehicle, a learning-based prediction-then-judgment model is proposed and designed to realize the prediction of the ICV's lane-change behavior. Experiments are implemented by using the modified dataset. From the experimental results, the lane-change probability value on the target lane in the truth of the lane-change behavior calculated by the designed HMM-based model is basically above 0.5, indicating that the model can make a more accurate judgment on the surrounding lane-change environment. The proposed learning-based prediction-then-judgment model has an accuracy of 99.32% for the prediction of lane-change behavior, and the accuracy of the lane-change detection algorithm in the model is 99.56%. The experimental results show that the proposed approach has a good performance in the prediction of lane-change behavior, which could effectively assist ICVs to change lanes safely.

## 1. Introduction

The intelligent and connected vehicle (ICV) [1] integrates modern communication and network technology and has environment perception, intelligent decision-making, and collaborative control functions. It can achieve safe, efficient, comfortable, and energy-saving driving and realize a new generation of vehicle that replaces humans [2].

Lane-change behavior detection and prediction plays an important role in the ICV technology. During the driving of the vehicle, the current driving environment may be misjudged due to the occlusion of surrounding vehicles or the driver's inattention, resulting in greater safety hazards. Therefore, the sensor and communication technology can assist the ICV to perceive and judge the surrounding environment and the state of the vehicle and combined with artificial intelligence technology can predict the lane-changing behavior, thereby improving the driving safety.

Many methods have been proposed for lane-change detection and prediction, in which the main technical means and data sources used can be summarized as trajectories, steering wheel, surrounding environment, driving style, computer vision and roadside LiDAR, etc., as shown in Table 1. Lane-change behavior detection methods based on trajectory data are proposed in Refs. [3–8], such as fuzzy logic [3], support vector machine (SVM) [4], long short-term memory network and convolutional neural network (LSTM-CNN) [5], maneuver classification [6], and hidden Markov model [7, 8]. Panichpapiboon and Leakkaw explore an approach to detect lane-change behavior using steering wheel angles extracted from the smart phone [9]. Zheng and Hansen propose an approach to detect lane-change behavior using the steering angle signal from CAN-bus [10]. Ali et al. propose a wavelet transform (WT)-based method to detect failed lane-changing attempts and used the random parameter binary logic model to study how the connected environment affects related parameters [11]. Woo et al. present a method to determine the driving styles and use the result to detect the lane-change behavior [12]. Nguyen et al. introduce a vision-based lane and vehicle detection approach for the lane-change assistant system [13]. Wang et al. present a method to detect lane-change behavior based on candidate lane markings [14]. Wei et al. develop a computer vision system to detect the lane-change behavior [15]. Cui et al. develop the methods to detect and predict lane-change behavior using vehicle trajectories from roadside LiDAR data [17]. Xu et al. present a V2X-based lane-change prediction model using vehicle trajectories [18]. Zhang and Fu present a lane-change intention detection method using motion parameters of the vehicle and surrounding vehicles [19]. Gao et al. introduce a lane-change behavior detection approach using multiple differing modality data [20]. Jin et al. present an optimal lane-change timing prediction model based on the driver's habits [21]. Huang et al. present a trajectory planning and control approach based on user preferences [22]. Xing et al. propose a driving pattern analysis and motion prediction system that determines the trajectory according to user's preference [23]. Xing et al. develop a driver intention inference system for highway lane-change maneuvers [16]. Xing et al. present a leading vehicle trajectory prediction approach that considers different driving styles [24].

In the above studies, different methods and models using different technical means and considering the influence of different characteristic parameters have been designed and proposed, fully verified, and achieved great results. However, few studies have simultaneously considered the effects of the vehicle, environment and driver, and the relationship between them when the ICV changes lanes. In this paper, a novel intelligent approach combines the driving state of the vehicle, the surrounding driving environment, and the driving style is proposed to predict the lane-change behaviors for ICVs. First, based on the learning of the driving habits of manual drivers, the current lane-change environment is judged according to the driving state of surrounding vehicles. If the current environment is suitable for lane change, then the vehicle driving state parameters are predicted, and the lane-change behavior detection method is proposed to judge the predicted value, so as to predict the lane-change behavior. The main contributions can be summarized as follows.

- According to the relevant characteristic parameters of the vehicle lane change, the NGSIM dataset is processed and analyzed, so that a modified dataset for the lane-change behavior prediction is established
- Based on the driving habits of manual drivers, a HMM-based model is designed to judge whether the current surrounding environment of the vehicle is suitable for the lane change
- Based on the analysis of lane-change behavior characteristics, a prediction model based on LSTM and lane-change feature judgment method is proposed to predict the state parameters of the vehicle and determine whether it will change lanes
- A novel approach based on intelligent and connected technology, which in combination with the driving style-based lane-change environment and the driving trajectory-related parameters of the vehicle and surrounding vehicles, is proposed and performed on the established dataset to predict the lane-change behavior of vehicles

The rest of the paper is organized as follows. In Section 2, the establishment process of the dataset is described. In Section 3, on the basis of fully analyzing the characteristics of lane-change behavior, the proposed approach to lane-change behavior prediction is introduced in detail.  Section 4 gives the experimental results and analysis of the proposed approach performed on the modified dataset. Section 5 concludes the research work and presents the future work.

### 1.1. Dataset Establishment

In this paper, the NGSIM dataset is processed to obtain the vehicle's trajectory and surrounding driving environment data, so as to combine the driver's driving style to build and verify the vehicle's lane-change prediction model.

### 1.2. Data Description

**Figure 1:**
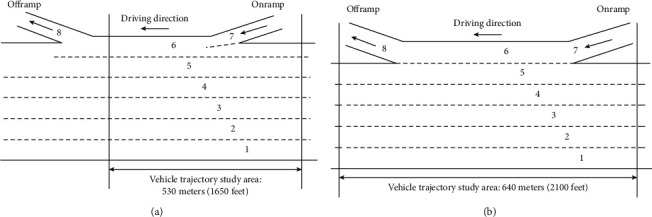
Study area description of the dataset.

The NGSIM is a dataset of different sections initiated by the United States Department of Transportation (US DOT) Federal Highway Administration (FHWA) [25]. In the NGSIM, I-80 and US-101 are the datasets collected in highway, which are studied in this paper. As shown in Figure 1, both I-80 and US-101 consist of five main lanes, one distribution lane, one on-ramp, and one off-ramp (the off-ramp of I-80 is not located within the study area). In I-80, the 1650-foot-long study area is divided into seven sub-areas by seven cameras to record the relevant data, while in US-101, the 2100-foot-long study area is divided into eight sub-areas by eight cameras. The dataset contains the trajectory data of all vehicles in the study area during the recorded time period.

### 1.3. Data Preprocessing

In order to analyze the characteristics of the lane-change behavior, characteristic parameters such as the coordinates and velocity of the vehicles are extracted from the NGSIM dataset. The coordinates of the ramp and the most adjacent lane have a large overlap, which will cause great interference to the study. Therefore, the data related to the ramp and the most adjacent lane are eliminated in the study. To further analyze the influence of the surrounding lane-change environment on lane-change behavior and the relationship between them, the distance between the vehicle and the front and rear vehicles on the current lane and adjacent lanes is calculated. The lateral speed of the vehicle is also calculated in order to predict the lane-change behavior. The complete data of 92 lane-changing vehicles, a total of 92932 frames, are finally screened out and processed; then a modified dataset is established.

**Figure 2:**
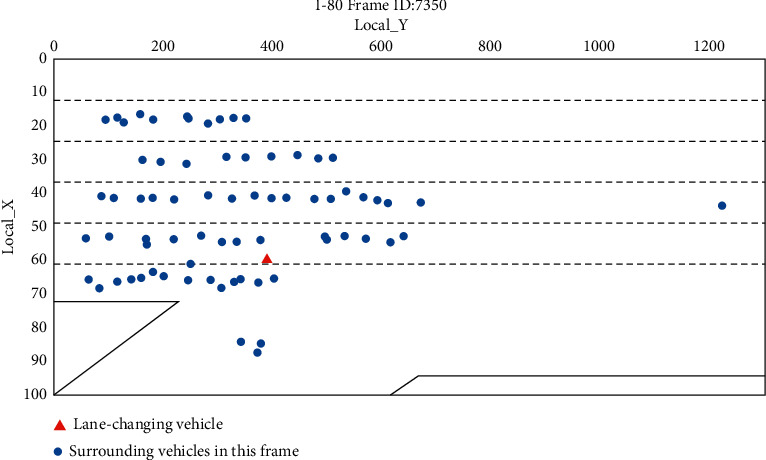
Schematic diagram of vehicle lane-changing state frame.

In the processed data, the surrounding lane-change environment at the time of a certain vehicle lane-change frame is shown in Figure 2. The range of lane coordinates calculated according to the data in the processed dataset is shown in Table 2.

## 2. Methodology

### 2.1. Analysis of Lane-Change Characteristics

**Figure 3:**
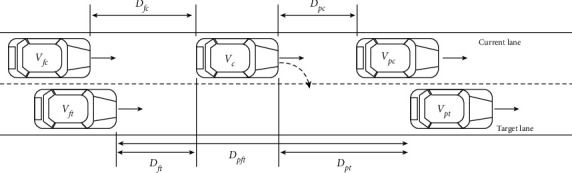
The typical lane-change scenario.

According to the study of Balal et al. [26], the main characteristics that affect drivers' lane change are *D*_*ft*_, *D*_*pft*_, *D*_*pt*_, *D*_*pc*_, and *V*_*c*_. Lane-change behaviors can be divided into left-lane change and right-lane change, so *D*_*ft*_, *D*_*pft*_, and *D*_*pt*_ can be divided into *D*_*fl*_, *D*_*fr*_, *D*_*pfl*_, *D*_*pfr*_, *D*_*pl*_, and *D*_*pr*_, which were defined in Table 3. The typical lane-change scenario taking the right-lane change as an example can be described in Figure 3.

### 2.2. Intelligent Prediction Approach

Based on the analysis of lane-change characteristics in real scene dataset, an intelligent prediction approach is proposed and established, in which the HMM-based model is used to judge the lane-change conditions, LSTM-based model is used to predict the current vehicle motion data that are suitable for change lanes, and then the designed lane-change detection algorithm is performed to complete the lane-change behavior prediction.

#### 2.2.1. HMM-Based Lane-Change Environment Judgment

HMM can be used to predict the probability of whether a vehicle changes lanes [27–29]. The vehicle and surrounding vehicles' driving state determines to a large extent whether the vehicle has the conditions for changing lanes. In this paper, based on the analysis of vehicle lane-change characteristics, eight parameters, *D*_*fl*_, *D*_*fr*_, *D*_*pfl*_, *D*_*pfr*_, *D*_*pl*_, *D*_*pr*_, *D*_*pc*_, and *V*_*c*_, are selected as observations to judge the surrounding lane-change environment.

As shown in Figure 3, in the HMM model, the eight continuous observation values, *D*_*fl*_, *D*_*fr*_, *D*_*pfl*_, *D*_*pfr*_, *D*_*pl*_, *D*_*pr*_, *D*_*pc*_, and *V*_*c*_, are used as observation vectors. In order to simplify the model and facilitate implementation in practical applications, the continuous values of the observation vector are divided into eight segments according to the importance of each observation vector's influence on lane-change behavior. The observation vector can be defined as follows:

(1) $$\begin{array}{c} V_{T}=O_{M}=\left[D_{ftT},D_{pftT},D_{ptT},D_{pcT},V_{cT}\right], \end{array}$$

where *V* *=* [*V*_1_, V_2_,…, *V*_*T*_*]* is the observation sequence, *T* is the sequence length, *O* *=* [*O*_1_, *O*_2_,…, *O*_*M*_*]* is the observation state, *M* is the distribution of the observable state, which is divided into 8 states according to the value of the observation vector and its importance [26], and *M*∈{1, 2, 3, 4, 5, 6, 7, 8}. The hidden states are the lane-change behaviors, including nonlane change, left-lane change, and right-lane change, which are represented as *H*_*1*_, *H*_*2*_, and *H*_*3*_, respectively.

**Figure 4:**
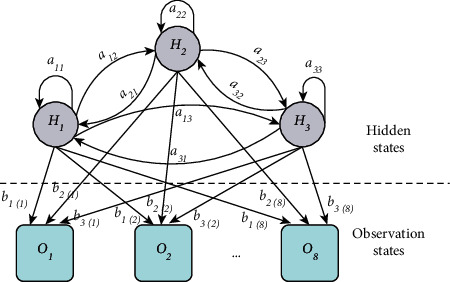
Schematic diagram of designed HMM.

As shown in Figure 4, the parameters of designed model can be defined as follows:

(2) $$\begin{array}{c} \lambda =\left(A,B,\pi \right), \end{array}$$

where *A* means the state transition probability matrix, in which *a*_*ij*_ is the probability of transition to state *h*_*j*_ at time *T* + 1 under the condition that time *T* is in state *h*_*i*_,

(3) $$\begin{array}{c} A={\left[a_{ij}\right]}_{3\times 3},\\ a_{ij}=P\left(i_{T+1}=h_{j}|i_{T}=h_{i}\right),\;i=1,2,3;\;j=1,2,3. \end{array}$$

*B* represents the observation probability matrix, in which *b*_*j(M)*_ is the probability of generating the observation *O*_*M*_ under the condition that time *T* is in state *h*_*j*_:

(4) $$\begin{array}{c} B={\left[b_{j\left(M\right)}\right]}_{3\times 8},\\ b_{j\left(M\right)}=P\left(V_{T}=O_{M}|i_{T}=h_{j}\right),\;M=1,2,...,8;\;j=1,2,3. \end{array}$$

*π* indicates the initial state probability distribution, in which *π*_*i*_ is the probability of being in state *h*_*i*_ at time *t* = 1:

(5) $$\begin{array}{c} \pi =\left(\pi_{i}\right),\\ \pi_{i}=P\left(i_{1}=h_{i}\right),\;i=1,2,3. \end{array}$$

In this paper, the dataset contains the observation sequence and the corresponding state sequence. Therefore, the supervised learning method can be used to estimate parameters of HMM. The maximum likelihood estimation method is used, and the specific method is as follows:

(1) Estimate the transition probability. Assume that the frequency of the sample at time *t* in state *i* and transition to state *j* at time *t* + 1 is *A*_*ij*_, then the estimation of state transition probability *a*_*ij*_ is as follows:
  (6) $$\begin{array}{c} \hat{a_{ij}}=\frac{A_{ij}}{\sum_{j=1}^{3}A_{ij}},\;i=1,2,3;\;j=1,2,3. \end{array}$$
(2) Estimate the probability of observation. Assume that the frequency of the sample state is *j* and the observation is *M* is *B*_*jM*_, then the estimation of the probability *b*_*j(M)*_ that the state is *j* and the observation is *M* is as follows:
  (7) $$\begin{array}{c} \hat{b_{j\left(M\right)}}=\frac{B_{jM}}{\sum_{M=1}^{8}B_{jM}},\;j=1,2,3;\;M=1,2,...,8. \end{array}$$
(3) Estimate the initial state probability. The estimate $\hat{\pi_{i}}$ of the initial state probability *π*_*i*_ is the frequency at which the initial state is *h*_*i*_ in the sample.

After the model parameters are determined, using the forward probability and the backward probability, given the model *λ* and the observation *O*, the probability of being in the state *h*_*i*_ at time *t* can be obtained:

(8) $$\begin{array}{c} \gamma_{t}=P\left(i_{t}=h_{i}|O,\lambda \right)=\frac{P\left(i_{t}=h_{i},O|\lambda \right)}{P\left(O|\lambda \right)}. \end{array}$$

From the definition of forward probability *α*_*t*_(*i*) and backward probability *β*_*t*_(*i*),

(9) $$\begin{array}{c} \alpha_{t}\left(i\right)\beta_{t}\left(i\right)=P\left(i_{t}=h_{i},O|\lambda \right). \end{array}$$

Then

(10) $$\begin{array}{c} \gamma_{t}\left(i\right)=\frac{\alpha_{t}\left(i\right)\beta_{t}\left(i\right)}{P\left(O|\lambda \right)}=\frac{\alpha_{t}\left(i\right)\beta_{t}\left(i\right)}{\sum_{j=1}^{3}\alpha_{t}\left(j\right)\beta_{t}\left(j\right)}. \end{array}$$

#### 2.2.2. LSTM-Based Vehicle Trajectory Prediction

**Figure 5:**
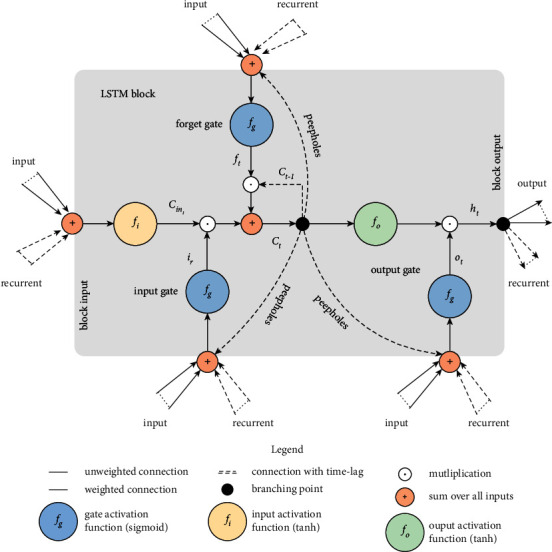
The structure of an LSTM block.

After judging the surrounding lane-change environment, the data suitable for lane change would be screened out to predict the lane-change behavior. An LSTM [30] model is designed to predict the current vehicle motion data. The structure of an LSTM block [31] is shown as Figure 5, in which *f*_*i*_ is the input activation function, *f*_*o*_ is the output activation function, and *f*_*g*_ is the gate activation function. At time *t*, *x*_*t*_ is the input, *h*_*t*_ is the hidden layer state, it is the output state of input gate, *f*_*t*_ is the output state of forget gate, and *o*_*t*_ is the output state of output gate, which can be expressed as follows:

(11) $$\begin{array}{c} i_{t}=f_{g}\left(w_{x_{i}}x_{t}+w_{h_{i}}h_{t-1}+b_{i}\right), \end{array}$$

(12) $$\begin{array}{c} {\text{f}}_{t}=f_{g}\left(w_{x_{f}}x_{t}+w_{h_{f}}h_{t-1}+b_{f}\right), \end{array}$$

(13) $$\begin{array}{c} o_{t}=f_{g}\left(w_{x_{o}}x_{t}+w_{h_{o}}h_{t-1}+b_{o}\right), \end{array}$$

where *w*_*x*_*i*__, *w*_*x*_*f*__, and *w*_*x*_*o*__ are the input weight matrices; *w*_*h*_*i*__, *w*_*h*_*f*__, and *w*_*h*_*o*__ who are the feedback weight matrices; and *b*_*i*_, *b*_*f*_, and *b*_*o*_ are the bias vectors.

The intermediate states at time *t* are as follows: the output state *C*_*in*_*t*__ corresponding to the input function, the output state *C*_*t*_ corresponding to the output function, and the output state *h*_*t*_ corresponding to the hidden layer.

(14) $$\begin{array}{c} C_{in_{t}}=f_{i}\left(w_{x_{c}}x_{t}+w_{h_{c}}h_{t-1}+b_{C_{in}}\right), \end{array}$$

where *w*_*x*_*c*__, *w*_*h*_*c*__, and *b*_*C*_*in*__ are the input weight matrix and the corresponding bias vector, respectively. *C*_*in*_*t*__, as the input function, the output state at time *t* will participate in the overall update of the input state at time *t* together with the output state *i*_*t*_ of the input gate at time *t*. As the output state of the input function at time *t*, *C*_*in*_*t*__ participates in the overall update of the input state at time *t* together with the output state *i*_*t*_ of the input gate at time *t*.

At time *t*, through the new input and state feedback at previous time, the entire LSTM unit is updated, including the update of *C*_*t*_ and *h*_*t*_:

(15) $$\begin{array}{c} C_{t}=f_{t}C_{t-1}+i_{t}C_{in_{t}}, \end{array}$$

(16) $$\begin{array}{c} h_{t}=o_{t}f_{o}\left(C_{t}\right). \end{array}$$

In the update process of each gate function and the output state of the entire unit, the key information in the input feature is retained and transferred through the forget gate function and the transfer of the state.

#### 2.2.3. The Lane-Change Behavior Prediction Approach

The predicted data with conditions for lane change are used to determine whether the current vehicle will change lanes through the lane-change detection algorithm, which can be described as shown in Algorithm 1. An optimal sampling interval length (*δ* sampling period) is obtained according to the data training, and then the velocity data on *x*-axis are used as input according to the obtained sampling interval length. In the process of lane-change detection, first, calculate all zero-crossing points in the input data and sort them by time; then, calculate the lateral velocity integral between all adjacent zero-crossing points; finally, train the calculation results based on the KNN [32, 33] method, and output lane-change detection result (the lane-change behavior category) and the start and end time of lane-change process.

**Figure 6:**
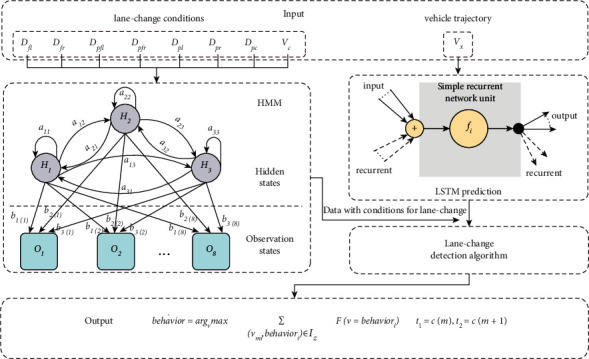
The structure of lane-change behavior prediction approach.

The structure of proposed multimodel fusion lane-change behavior prediction approach can be described as shown in Figure 6. First, the surrounding environment parameters related to the lane-change condition are used to judge the current lane-change condition through the HMM-based model. Then, the vehicle trajectory data which meet lane-change conditions are predicted by the LSTM-based model. Finally, the lane-change behavior is predicted by the proposed lane-change detection algorithm; the predicted lane-change behavior and the start and end time of lane-change process are output.

## 3. Results and Analysis

### 3.1. Evaluation Metrics

When evaluating the prediction results, the confusion matrix definition of the prediction results is shown in Table 4. Accuracy, precision, recall, and *F*1 value are usually used as evaluation indicators [34] for learning-based classification and prediction models. Among them, the accuracy represents the proportion of the sample size correctly classified in the total sample size, which can be defined as follows:

(17) $$\begin{array}{c} ACC=\frac{TP+TN}{TP+TN+FN+FP}. \end{array}$$

Precision (*P*), which indicates the proportion of samples with the correct class label among the samples of a particular class found by the classifier, can be defined as follows:

(18) $$\begin{array}{c} P=\frac{TP}{TP+FP}. \end{array}$$

The recall (*R*) represents the classifier's ability to find samples of a certain category, which can be defined as follows:

(19) $$\begin{array}{c} R=\frac{TP}{TP+FN}. \end{array}$$

The *F*1 value is a comprehensive index that considers the balance between precision and recall, which can be defined as follows:

(20) $$\begin{array}{c} F1=\frac{2\times P\times R}{P+R}. \end{array}$$

The closer the *F*1 value is to 1, the better the effect.

### 3.2. Experimental Results

The proposed prediction approach, including HMM-based lane-change condition judgment, LSTM-based vehicle lane change-related parameter prediction, and lane-change detection algorithm, is trained and tested on the established modified dataset to evaluate the performance.

#### 3.2.1. Performance of HMM-Based Model

In order to verify the judgment performance of the HMM-based model on the lane-change environment, parameters related to lane change, including *D*_*fl*_, *D*_*fr*_, *D*_*pfl*_, *D*_*pfr*_, *D*_*pl*_, *D*_*pr*_, *D*_*pc*_, and *V*_*c*_, are processed and then trained and tested. The results show that at all lane-change times, the lane-change probability of target lane is basically above 0.5. At the moment when the vehicle does not change lanes, some lane-change environments meet the lane-change conditions, and some do not. Therefore, the designed model can screen out the moments that do not meet the lane-change conditions and improve the prediction accuracy.

**Figure 7:**
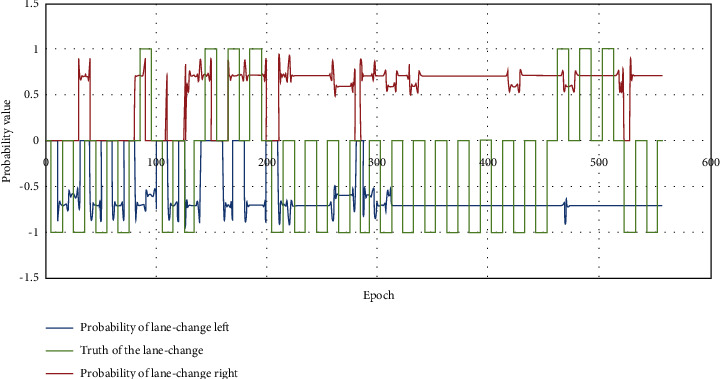
Schematic fragment of the designed HMM-based judgment result of lane-change condition.

The schematic fragment of the designed HMM-based judgment result of lane-change condition is shown in Figure 7. In the figure, the data of the green line represent the truth of the lane-change behavior (1 means right-lane change, −1 means left-lane change, 0 means nonlane change), the data of the red line indicate the probability of the right-lane change calculated by the designed model, while the data of the blue line denote the probability of the left-lane change calculated by the designed model. It can be seen that the calculated right-lane-change probability at the time of right-lane change and the left-lane-change probability at the time of left-lane change in the figure are all greater than 0.5, which meets the lane-change conditions.

#### 3.2.2. Performance of LSTM-Based Model

**Figure 8:**
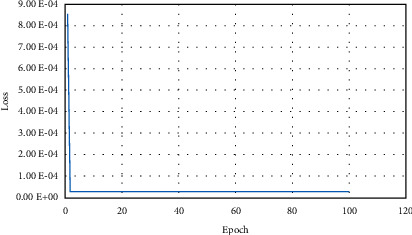
The loss curve of LSTM.

In order to predict the lane-change behavior of the ICV, the LSTM-based model is designed to predict the lane-change-related motion data (lateral velocity) at the next moment. The dataset is divided into training set and test set at a ratio of 2 to 1 to verify the performance of the designed model. The loss curve of the training process is shown in Figure 8, in which the loss value is stable at around 2.32E-05.

**Figure 9:**
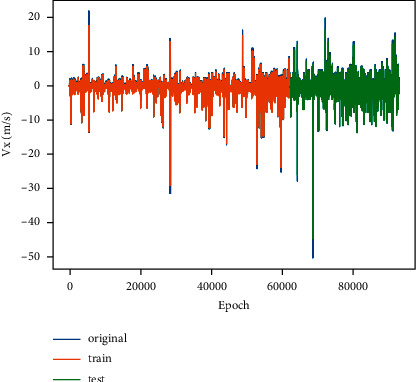
The prediction result of designed LSTM.

The prediction result of designed LSTM is shown in Figure 9, in which the blue line represents original data and yellow line and green line indicate the prediction result of the training set and the test set, respectively. The root mean square error (RMSE) of the prediction is 0.37 m/s for the training set and 0.68 m/s for the test set. From the prediction results in the figure, it can be found that the overall prediction error of the designed model is small, and the prediction error is greater when the data have large and sudden changes than when the data are flat. The maximum prediction error of the dataset is 5.5668 m/s (the original data is 50.0055 m/s).

#### 3.2.3. Performance of the Detection Algorithm

**Figure 10:**
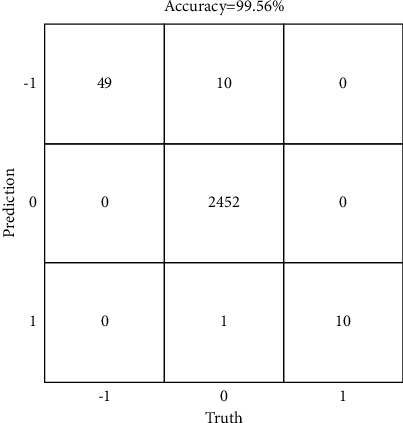
The confusion matrix of detection result.

The designed lane-change behavior detection algorithm was performed on the established dataset to verify its detection effect on lane-change behavior. The dataset is divided into training set and test set at a ratio of 2 to 1, the experimental result of detection is shown in Table 5, and the confusion matrix of it is shown in Figure 10.

It can be seen from the experimental result of detection that the *P* of left-lane-change detection has reached 100% and *R* of it is 83.05%, the *P* of right-lane-change detection is 90.91% and *R* of it is 100%, while *P* and *R* of nonlane change are 99.55% and 100%, respectively. The *F1* values of left-lane change, nonlane change, and right-lane change are 90.74%, 99.77%, and 95.24%, respectively.

From the confusion matrix of detection result, it can be found that the accuracy of the detection algorithm is 99.56%. Among them, 10 samples in nonlane change are detected as left-lane change, and 1 sample is detected as right-lane change, while no lane-change behavior is detected as nonlane change and there is no error detection between left-lane change and right-lane change, which shows that the proposed detection algorithm could accurately detect lane-change information for the safe lane change of ICV.

**Figure 11:**
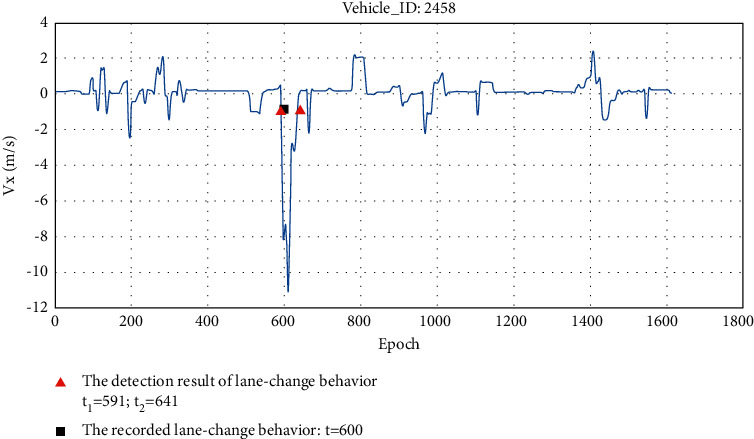
The detection result of vehicle 2458.

Taking entire driving process of vehicle 2458 as an example, the detection result is shown in Figure 11. It can be seen from the figure that vehicle 2458 made a lane change at *t* = 600 during the whole process, and its lateral velocity has an obvious acceleration process. The designed detection algorithm accurately detects the lane-change behavior and calculates the lane-change process that is between *t*_*1*_ = 591 and *t*_*2*_ = 641.

#### 3.2.4. Performance of the Lane-Change Behavior Prediction Approach

**Figure 12:**
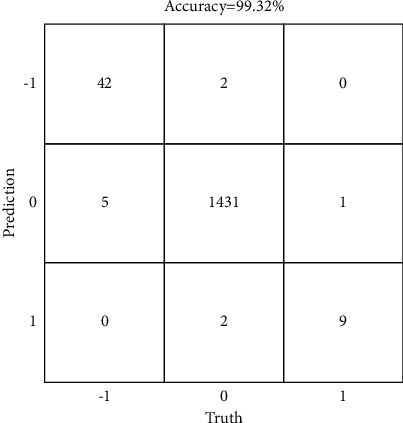
The confusion matrix of prediction result.

Finally, the proposed prediction approach is performed on the established dataset to verify the effect of the approach. The proposed lane-change behavior detection algorithm is performed on the filtered prediction data that meets the lane-change conditions. The dataset is also divided into training set and test set at a ratio of 2 to 1, the experimental result of prediction is shown in Table 6, and the confusion matrix of prediction result is shown in Figure 12.

From the experimental result of prediction, it can be seen that *P* of left-lane-change detection is 89.36% and *R* of it is 95.45%, the *P* of right-lane-change detection is 90.00% and *R* of it is 81.82%, while *P* and *R* of nonlane change are 99.72% and 99.65%, respectively. The *F*1 values of left-lane change, nonlane change and right-lane change are 92.31%, 99.65%, and 85.71%, respectively.

It can be found from the confusion matrix of prediction result that the accuracy of the prediction approach is 99.32%. Among them, 2 samples in nonlane change are predicted as left-lane change, and 2 samples are predicted as right-lane change. 5 samples in left-lane change are predicted as nonlane change, with a precision of 89.36%, and 1 sample in right-lane change is predicted as nonlane change, with a precision of 90.00%, while there is no error detection between left-lane change and right-lane change. The experimental result shows that the proposed prediction approach could effectively provide vehicle lane-change information to assist the ICV in safe lane change.

**Figure 13:**
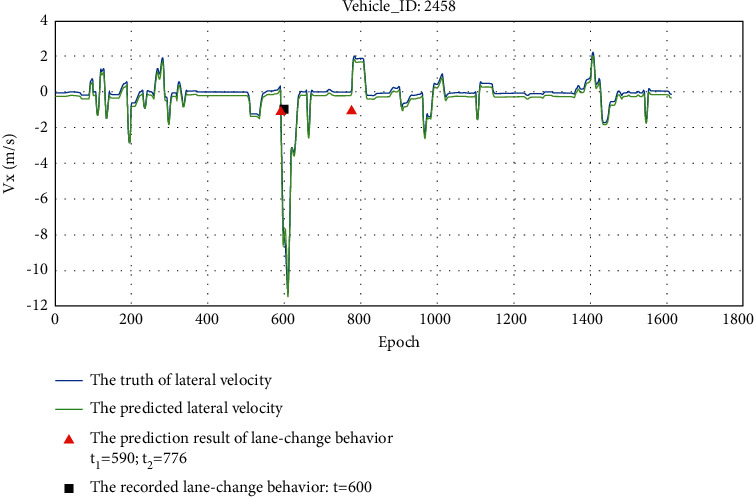
The prediction result of vehicle 2458.

Taking the entire driving process of vehicle 2458 as an example, the prediction result is shown in Figure 13. It can be seen that the predicted lateral velocity and the truth of lateral velocity are basically consistent in value and trend. The designed prediction approach accurately predicts the lane-change behavior and calculates the lane-change process that is between *t*_*1*_ = 590 and *t*_*2*_ = 776. The predicted time interval of the lane-change process is longer than that calculated by the detection algorithm, which is because that when the lateral velocity value fluctuates around 0, the predicted lateral velocity value fluctuates slightly and is less than 0. The prediction approach can still accurately predict the lane-change behavior and the time interval of lane change.

## 4. Conclusions

The paper proposed a novel intelligent approach to lane-change behavior prediction for ICVs, which combines the surrounding lane-change environment and the vehicle's own motion parameters. A modified dataset is established based on the NGSIM dataset, and then the proposed approach is trained and tested. From the experimental results, the HMM-based model can make relatively accurate judgments on the lane-change environment, and its calculated lane-change probability at the time of lane change is above 0.5. The prediction RMSE of the vehicle lateral speed by the LSTM-based model is 0.37 m/s for the training set and 0.68 m/s for the test set. The proposed lane-change detection algorithm has an accuracy of 99.56% on the established dataset and can accurately calculate the time interval of the vehicle lane change. On the basis of the fusion of the above models and algorithm, the proposed intelligent prediction approach is completed, result shows that the accuracy of the prediction approach on the established dataset is 99.32%, and the time interval of the vehicle lane change can be calculated accurately. The experimental result indicates that the proposed prediction approach could effectively provide vehicle lane-change information to assist the ICV in safe lane-change and has the potentials for application in actual intelligent and connected environment for ICVs.

Since the proposed approach is postprocessing with measured data, its real-time performance in practical applications needs to be further verified. In future work, the proposed approach can be deployed on mobile terminals for real-time testing, and its accuracy and real-time performance can be further improved.

## Figures and Tables

**Algorithm 1:**
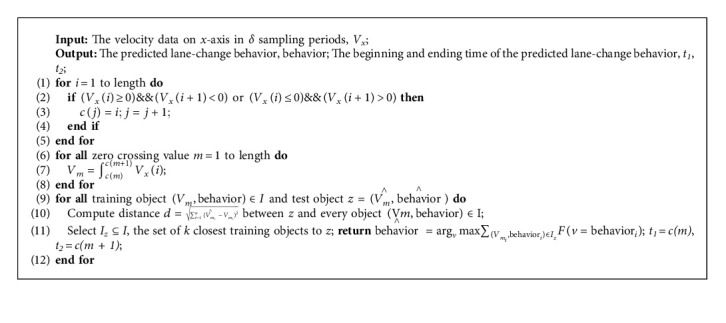
Lane-change detection algorithm.

**Table 1:** Comparison of lane-change detection or prediction approaches.

	Trajectories	Steering wheel	Surrounding environment	Driving style	Computer vision	Roadside LiDAR
[3–8]	**√**					
[9, 10]		**√**				
[11]			**√**			
[12]				**√**		
[13–16]					**√**	
[17]						**√**
[18, 19]	**√**		**√**			
[20]	**√**				**√**	
[21]			**√**	**√**		
[22–24]	**√**			**√**		
Proposed approach	**√**		**√**	**√**		

**Table 2:** The range of lane coordinates.

Coordinate (feet)	Lane ID			
Dataset	1	2	3	4
I-80	0.21–12.29	11.90–24.38	23.74–36.10	35.63–48.42
US-101	1.46–13.33	11.51–24.45	23.41–35.68	33.58–46.29

Coordinate (feet)	Lane ID			
Dataset	5	6	7	8
I-80	47.83–60.42	59.52–82.48	72.65–96.75	
US-101	44.99–61.30	57.19–69.60	57.38–72.60	58.04–72.92

**Table 3:** Definition of selected parameters.

Parameters	Definition
*D*_*fl*_	The distance between the current vehicle and the following vehicle in the left/right lane
*D*_*fr*_	
*D*_*pfl*_	The distance between the preceding vehicle and the following vehicle in the left/right lane
*D*_*pfr*_	
*D*_*pl*_	The distance between the current vehicle and the preceding vehicle in the left/right lane
*D*_*pr*_	
*D*_*pc*_	The distance between the current vehicle and the preceding vehicle in the current lane The velocity of current vehicle
*V*_*c*_	

**Table 4:** Definition of the prediction confusion matrix.

Predicted		
Truth	Positive	Negative
True	TP	TN
False	FP	FN

**Table 5:** Experimental result of detection algorithm.

Metrics			
Category	*P* (%)	*R* (%)	*F*1 (%)
Left-lane change	100	83.05	90.74
Nonlane change	99.55	100	99.77
Right-lane change	90.91	100	95.24

**Table 6:** Experimental result of prediction approach.

Metrics			
Category	*P* (%)	*R* (%)	*F*1 (%)
Left-lane change	89.36	95.45	92.31
Nonlane change	99.72	99.58	99.65
Right-lane change	90.00	81.82	85.71

## Data Availability

The modified NGSIM data used to support the findings of this study are available from the corresponding author upon request.
